# Overexpression of heterogeneous nuclear ribonucleoprotein F stimulates renal *Ace-2* gene expression and prevents TGF-β1-induced kidney injury in a mouse model of diabetes

**DOI:** 10.1007/s00125-015-3700-y

**Published:** 2015-08-01

**Authors:** Chao-Sheng Lo, Yixuan Shi, Shiao-Ying Chang, Shaaban Abdo, Isabelle Chenier, Janos G. Filep, Julie R. Ingelfinger, Shao-Ling Zhang, John S. D. Chan

**Affiliations:** 1 Centre de recherche, Centre hospitalier de l’Université de Montréal (CRCHUM) – Tour Viger Pavillon R, Université de Montréal, 900 Saint-Denis Street, Montreal, QC H2X 0A9 Canada; 2 Research Centre, Maisonneuve-Rosemont Hospital, Université de Montréal, Montreal, QC Canada; 3 Pediatric Nephrology Unit, Massachusetts General Hospital, Harvard Medical School, Boston, MA USA

**Keywords:** ACE-2, Akita mice, Angiotensinogen, Diabetes, Heterogeneous nuclear ribonucleoprotein F, Hypertension, Renal fibrosis, TGF-β1

## Abstract

**Aims/hypothesis:**

We investigated whether heterogeneous nuclear ribonucleoprotein F (hnRNP F) stimulates renal ACE-2 expression and prevents TGF-β1 signalling, TGF-β1 inhibition of *Ace-2* gene expression and induction of tubulo-fibrosis in an Akita mouse model of type 1 diabetes.

**Methods:**

Adult male Akita transgenic (Tg) mice overexpressing specifically hnRNP F in their renal proximal tubular cells (RPTCs) were studied. Non-Akita littermates and Akita mice served as controls. Immortalised rat RPTCs stably transfected with plasmid containing either rat *Hnrnpf* cDNA or rat *Ace-2* gene promoter were also studied*.*

**Results:**

Overexpression of hnRNP F attenuated systemic hypertension, glomerular filtration rate, albumin/creatinine ratio, urinary angiotensinogen (AGT) and angiotensin (Ang) II levels, renal fibrosis and profibrotic gene (*Agt*, *Tgf-β1*, TGF-β receptor II [*Tgf-βrII*]) expression, stimulated anti-profibrotic gene (*Ace-2* and *Ang 1–7* receptor [*MasR*]) expression, and normalised urinary Ang 1–7 level in Akita *Hnrnpf*-Tg mice as compared with Akita mice. In vitro, hnRNP F overexpression stimulated *Ace-2* gene promoter activity, mRNA and protein expression, and attenuated *Agt*, *Tgf-β1* and *Tgf-βrII* gene expression. Furthermore, hnRNP F overexpression prevented TGF-β1 signalling and TGF-β1 inhibition of *Ace-2* gene expression.

**Conclusions/interpretation:**

These data demonstrate that hnRNP F stimulates *Ace-2* gene transcription, prevents TGF-β1 inhibition of *Ace-2* gene transcription and induction of kidney injury in diabetes. HnRNP F may be a potential target for treating hypertension and renal fibrosis in diabetes.

**Electronic supplementary material:**

The online version of this article (doi:10.1007/s00125-015-3700-y) contains peer-reviewed but unedited supplementary material, which is available to authorised users.

## Introduction

Diabetic nephropathy (DN), a leading cause of end-stage renal disease (ESRD), accounts for ∼50% of all ESRD cases [1, 2]. While glomerulopathy is a hallmark of early renal injury in DN [3], tubulointerstitial fibrosis and tubular atrophy are major features of late-stage DN and are closely associated with loss of renal function [4–7]. The mechanisms underlying tubulointerstitial fibrosis, however, are incompletely understood. TGF-β1 is considered to be the most potent inducer of fibrogenesis [8]. Indeed, patients and animal models with type 1 or 2 diabetes have significantly elevated serum and urinary TGF-β1 levels [9–11] as well as heightened *TGF-β1* mRNA and protein expression in glomeruli and the tubulointerstitium [12–16].

We previously reported that high glucose milieu enhances expression of angiotensinogen (AGT, the sole precursor of all angiotensins) through generation of reactive oxygen species (ROS) in cultured rat renal proximal tubular cells (RPTCs) [17, 18]. Rat AGT overexpression in RPTCs leads to hypertension, albuminuria and RPTC hypertrophy, and enhances TGF-β1 expression in diabetic AGT-transgenic (Tg) mice [19, 20]. Conversely, RPTC-selective overexpression of catalase or pharmacological blockade of the renin–angiotensin system (RAS) attenuates hypertension, ROS generation, kidney injury and normalised RPTC ACE-2 expression in mouse models of diabetes [21–24]. Taken together, these observations indicate that oxidative stress-induced upregulation of AGT expression and downregulation of ACE-2 expression in RPTCs, resulting in higher angiotensin (Ang)II/Ang 1–7 ratio, may be key determinants of development of hypertension and nephropathy in diabetes.

We reported that insulin inhibits high glucose stimulation of rat renal *Agt* gene expression via two nuclear proteins—heterogeneous nuclear ribonucleoproteins F and K (hnRNP F, hnRNP K)—that interact with the insulin-responsive element (IRE) in the *Agt* gene promoter [25–28], and that hnRNP F overexpression in RPTCs inhibits *Agt* gene expression and kidney hypertrophy in Akita *Hnrnpf*-Tg mice [29]. Here, we report that overexpression of hnRNP F stimulates *Ace-2* gene transcription and suppresses profibrotic gene (*Tgf-β1*, *Tgf-βrII*) expression in RPTCs of Akita *Hnrnpf*-Tg mice. We have confirmed these changes by in vitro studies in rat RPTCs. We also show that hnRNP F overexpression prevents TGF-β1 signalling and inhibition of *Ace-2* gene expression in RPTCs. Finally, we identified the putative DNA response elements (REs) in the *Ace-2* gene promoter that are responsive to hnRNP F and TGF-β1.

## Methods

### Chemicals and constructs

Active human recombinant TGF-β1 was obtained from R&D Systems (Minneapolis, MN, USA). SB431542 (a TGF-β receptor I [RI] inhibitor) and other chemicals were purchased from Sigma-Aldrich (Oakville, ON, Canada). The antibodies used in the present study are listed in electronic supplementary material (ESM) Table 1. The pKAP2 plasmid containing the kidney-specific androgen-regulated protein (KAP) promoter was a gift from C. D. Sigmund (University of Iowa, Iowa City, IA, USA) [30]. Full-length rat *Hnrnpf* cDNA fused with HA tag (encoding amino acid residues 98–106 [YPYDVPDYA] of human influenza virus hemagglutinin) was inserted into pKAP2 plasmid at the NotI site at both 5′ and 3′ termini [25, 29]. pGL4.20 vector containing Luciferase reporter was obtained from Promega (Sunnyvale, CA, USA). Rat *Ace-2* gene promoter (N-1,091/+83) was cloned from rat genomic DNA with specific primers (ESM Table 2), as described by Milsted et al [31] and then inserted into pGL4.20 plasmid at HindIII and KpnI restriction sites. Scrambled Silencer Negative Control no. 1 small interfering RNA (siRNA) and *Hnrnpf* siRNA were bought from Ambion (Austin, TX, USA). QuickChange II Site-Directed Mutagenesis Kit and LightShift Chemiluminescent electrophoretic mobility shift assay (EMSA) Kit were procured from Agilent Technologies (Santa Clara, CA, USA) and Thermo Scientific (Life Technologies, Burlington, ON, Canada), respectively. The primer biotin-labelling kit was purchased from Integrated DNA Technologies (Coralville, IA, USA).

### Physiological studies

Adult male heterozygous Akita mice (*Mus musculus*) with a mutated *Ins2* gene (C57BL6-*Ins2* ^Akita^/J) were purchased from Jackson Laboratories (Bar Harbor, ME, USA: http://jaxmice.jax.org). Akita Tg mice (C57Bl/6 background) overexpressing rat hnRNP F-HA in RPTCs (line 937) were created in our laboratory (by J. S. D. Chan) [29]. Male adult non-Tg and non-Akita littermates served as wild-type (WT) controls, and were tested along with *Hnrnpf*-Tg, Akita and Akita *Hnrnpf*-Tg mice. All animals were housed individually in metabolic cages for 24 h before euthanasia at age 20 weeks. All animals were fed standard mouse chow and water ad libitum. Animal care and procedures were approved by the CRCHUM Animal Care Committee and followed the Principles of Laboratory Animal Care (NIH publication no. 85-23, revised 1985: http://grants1.nih.gov/grants/olaw/references/phspol.htm).

Blood glucose levels, following 4–5 h fasting, were determined with an Accu-Chek Performa System (Roche Diagnostics, Laval, QC, Canada). Body weight (BW) was recorded. Urine was collected and assayed for albumin/creatinine ratio (ACR) by enzyme-linked immunosorbent assays (Albuwell and Creatinine Companion, Exocell, Philadelphia, PA, USA).

GFR was measured as described by Qi et al [32] as recommended by the Animal Models of Diabetic Complications Consortium (www.diacomp.org) with fluorescein isothiocyanate inulin [23, 28, 33].

Kidneys were removed immediately after GFR measurement, decapsulated and weighed. The left kidneys were processed for histology and immunostaining, and right renal cortices were harvested for renal proximal tubules (RPTs) isolation by Percoll gradient centrifugation [23, 24, 28, 29]. Aliquots of freshly isolated RPTs from individual mice were immediately processed for total RNA and protein isolation.

### Immunohistochemical staining

Immunohistochemical staining was performed by the standard avidin-biotin-peroxidase complex method in four to five sections (4 μm thick) per kidney and three mouse kidneys per group (ABC Staining System; Santa Cruz Biotechnology [Santa Cruz, CA, USA]) [23, 24, 28, 29]. Staining was analysed under light microscopy by two independent, blinded observers. The collected images were assessed by National Institutes of Health Image J software (http://rsb.info.nih.gov/ij/) [23, 24, 28, 29].

### Urinary AGT, Ang II and Ang 1–7 measurement

Mouse urinary AGT, Ang II and Ang 1–7 levels were analysed by ELISA (Immuno-Biological Laboratories, IBL America, Minneapolis, MN, USA) and normalised by urinary creatinine levels as described [23, 24, 28, 29, 34].

### Cell culture

Immortalised rat RPTCs (passages 12–18) [35] were cultured in 5 mmol/l d-glucose DMEM containing 5% FBS until they reached 60–70% confluence. The media were then changed to serum-free DMEM, ensuring that endogenously secreted TGF-β1 would not interfere in the assay. After 45 min preincubation, active human recombinant TGF-β1 [36] (0 to 10 ng/ml) was added (considered as time 0 h) and incubated for various time periods up to 24 h. In separate experiments, RPTCs were incubated for 24 h in serum-free medium in the presence or absence of TGF-β1± various concentrations of SB431542.

### Real-time quantitative PCR

*Hnrnpf*, *Ace*, *Ace-2*, *MasR*, *Tgf-β1*, *Tgf-βrI*, *Tgf-βrII*, collagen type IV, collagen type I, fibronectin 1 and β-actin mRNA expression levels in RPTs were quantified by real-time quantitative PCR (RT-qPCR) with forward and reverse primers (ESM Table 2) [23, 24, 28, 29].

### Western blotting

Western blotting (WB) was performed as described previously [23, 24, 28, 29]. The relative densities of hnRNP F, ACE, ACE-2, Ang 1–7 receptor (MasR), TGF-β1, TGF-β RI, TGF-β RII, fibronectin 1, p-Smad2/3, Smad2/3 and β-actin bands were quantified by computerised laser densitometry (ImageQuant software, version 5.1; Molecular Dynamics, Sunnyvale, CA, USA).

### Statistical analysis

The data are expressed as means ± SEM. Statistical analysis was performed by the Student’s *t* test or one-way analysis of variance and the Bonferroni test as appropriate provided by Graphpad Software, Prism 5.0 (www.graphpad.com/prism/Prism.htm). A value of *p* ≤ 0.05 was considered to be statistically significant.

## Results

### Physiological variables in Akita and Akita *Hnrnpf*-Tg mice

Table 1 documents significantly higher blood glucose levels in Akita compared with WT mice and *Hnrnpf*-Tg mice. Overexpression of hnRNP F had no effect on blood glucose levels in Akita *Hnrnpf*-Tg mice. Systolic BP (SBP), kidney weight (KW)/BW and KW/tibial length (TL) ratios, GFR and ACR were all elevated in Akita mice, compared with both WT controls and *Hnrnpf*-Tg mice. HnRNP F overexpression in RPTCs markedly attenuated these changes in diabetic Akita *Hnrnpf*-Tg mice. Furthermore, Akita mice exhibited elevated urinary AGT and Ang II levels, parallel with decreased Ang 1–7 levels, compared with WT mice. HnRNP F overexpression partially reduced urinary AGT and Ang II levels, whereas it completely normalised urinary Ang 1–7 levels—a novel finding.

**Table 1:** Physiological measurements

	WT	*Hnrnpf*-Tg	Akita	Akita *Hnrnpf*-Tg
Blood glucose (mmol/l)	10.8 ± 0.64	11.2 ± 0.67	34.5 ± 0.71***	35.1 ± 0.79***
SBP (mmHg)	110.7 ± 2.71	113.8 ± 2.67	133.4 ± 2.59**	121.5 ± 3.52**^††^
KW (mg)	398.7 ± 16.01	396.9 ± 1,936	550.0 ± 27.60**	432.7 ± 21.97^*†^
BW (g)	38.3 ± 1.41	34.9 ± 1.3	26.4 ± 0.85**	25.0 ± 0.45**
TL (mm)	22.6 ± 0.16	22.7 ± 0.21	22.3 ± 0.36	22 ± 0.13
KW/BW ratio	10.5 ± 0.57	11.3 ± 0.38	20.7 ± 0.54**	16.6 ± 1.15**^†^
KW/TL ratio	17.6 ± 0.67	17.4 ± 0.78	24.6 ± 1.14**	18.7 ± 1.25^†^
GFR (μl min^−1^ g^−1^)	7.3 ± 0.44	8.3 ± 0.39	19.8 ± 1.61**	16.2 ± 0.85**^†^
Urinary ACR (mg/mmol)	1.8 ± 0.33	1.8 ± 0.35	13.6 ± 3.25**	5.8 ± 1.07*^†^
Urinary AGT/Cre ratio (pmol/μmol)	1,418 ± 242.4	1,439 ± 137.5	4,512 ± 753.6**	2,804 ± 204.7**^†^
Urinary Ang II/Cre ratio (pmol/μmol)	19.56 ± 6.065	19.5 ± 7.964	299.38 ± 89.06**	133.05 ± 12.68**^†^
Urinary Ang 1–7/Cre ratio (pmol/μmol)	17.97 ± 1.807	18.30 ± 2.019	10.99 ± 0.734^*^	17.45 ± 1.238^†^

All data are expressed as means ± SEM

*p < 0.05, **p < 0.01 vs WT, ^†^ *p* < 0.05, ^††^ *p* < 0.01 vs Akita mice

### Effect of hnRNP F overexpression on AGT, ACE, ACE-2 and MasR expression in Akita *Hnrnpf*-Tg mouse kidneys

**Fig. 1:**
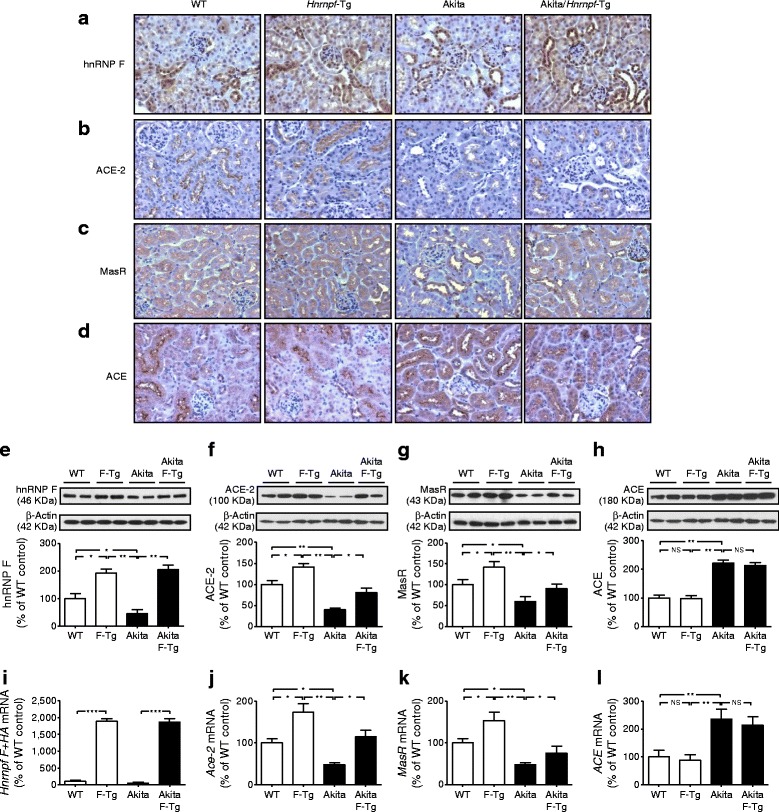
hnRNP F overexpression upregulates ACE-2 and MasR expression in mouse kidneys. Immunohistochemical staining of hnRNP F (**a**), ACE-2 (**b**), MasR (**c**) and ACE (**d**) expression in kidney sections (×200); WB (**e**–**h**) and RT-qPCR (**i**–**l**) of their respective protein and mRNA levels in freshly isolated RPTs from non-diabetic WT controls, *Hnrnpf*-Tg mice (F-Tg), diabetic Akita mice and Akita *Hnrnpf*-Tg mice (Akita F-Tg) at week 20. Values are means + SEM corrected to β-actin, *n* = 6. *p < 0.05; **p < 0.01; ***p < 0.001

Immunostaining revealed that HnRNP F (Fig. 1a) was overexpressed in RPTCs of *Hnrnpf*-Tg and Akita *Hnrnpf*-Tg mice compared with WT and Akita mice, respectively. ACE-2 (Fig. 1b) and MasR (Fig. 1c) expression was decreased in Akita mice compared with WT controls and normalised in Akita *Hnrnpf*-Tg mice. RPTC ACE (Fig. 1d) expression did not differ between WT and *Hnrnpf*-Tg mice, whereas ACE expression was significantly higher in Akita mice than in WT controls and was not normalised in Akita *Hnrnpf*-Tg mice. WB and RT-qPCR for hnRNP F, ACE-2, MasR and ACE protein and their mRNA levels (Fig. 1e–l, respectively) confirmed these observations.

### Effect of hnRNP F overexpression on TGF-β1, TGF-β RII and TGF-β RI expression in Akita *Hnrnpf*-Tg mouse kidneys

**Fig. 2:**
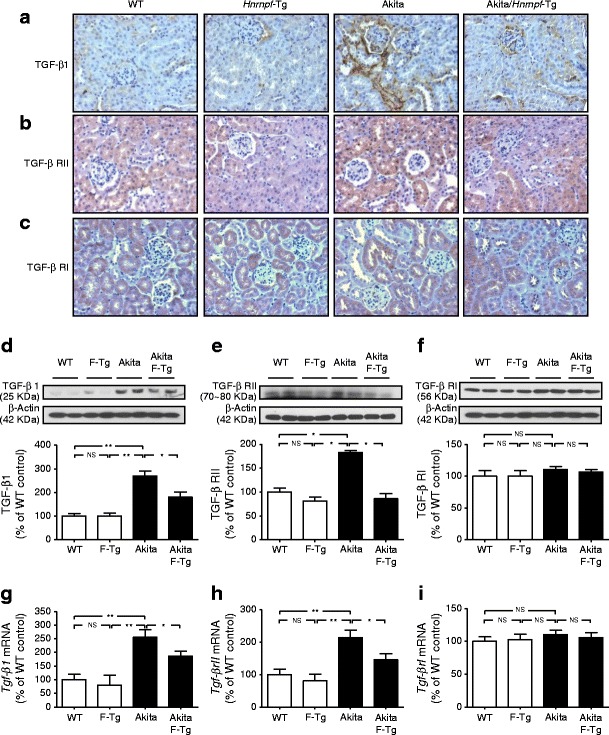
hnRNP F overexpression attenuates TGF-β1 and TGF-β RII expression in mouse kidneys. Immunohistochemical staining of TGF-β1 (**a**), TGF-β RII (**b**) and TGF-β RI (**c**) expression in kidney sections (×200), WB (**d**–**f**) and RT-qPCR (**g**–**i**) of their respective protein and mRNA levels in freshly isolated RPTs from non-diabetic WT controls, *Hnrnpf*-Tg (F-Tg) mice, diabetic Akita mice and Akita *Hnrnpf*-Tg mice (Akita F-Tg) at week 20. Values are means + SEM corrected to β-actin, *n* = 6. *p < 0.05; **p < 0.01

Immunostaining of TGF-β1 (Fig. 2a) and TGF-β RII (Fig. 2b), WB of TGF-β1 (Fig. 2d) and TGF-β RII expression (Fig. 2e), and RT-qPCR of *Tgf-β1* (Fig. 2g) and *Tgf-βrII* (Fig. 2h) mRNA expression showed significantly higher TGF-β1 and TGF-β RII expression in RPTCs of Akita mice than in WT controls and *Hnrnpf*-Tg mice, and they were attenuated in Akita *Hnrnpf*-Tg mice. In contrast, TGF-β RI expression was similar in all groups studied (Fig. 2c,f,i).

### HnRNP F overexpression suppresses renal fibrosis in Akita *Hnrnpf*-Tg mice

**Fig. 3:**
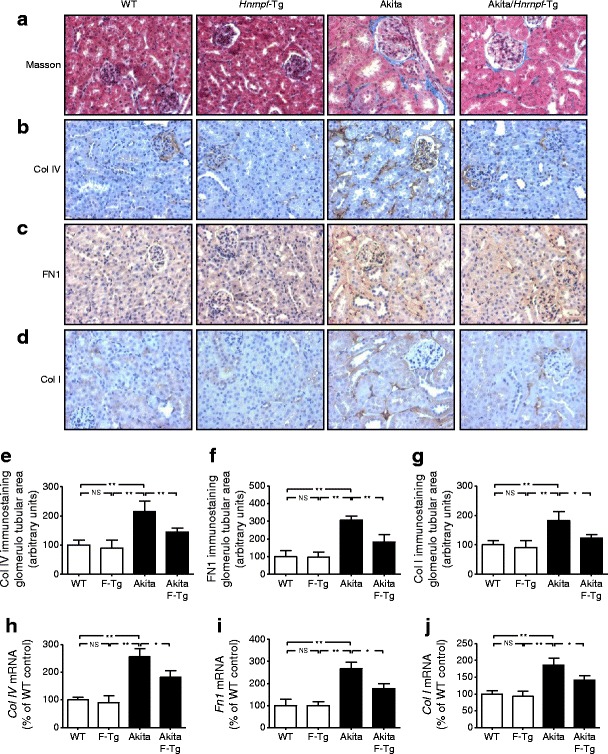
hnRNP F overexpression attenuates renal fibrosis and profibrotic gene expression in mouse kidneys. Masson’s trichrome staining (**a**), immunostaining of collagen IV (Col IV) (**b**), fibronectin 1 (FN1) (**c**) and collagen I (Col I) (**d**) expression in kidney sections (×200); semiquantitative analysis of immunostained collagen IV (**e**), fibronectin 1 (**f**) and collagen I (**g**), and RT-qPCR of collagen IV (also known as *Col4a1*) (**h**), *Fn1* (**i**) and collagen I (also known as *Col1a1*) (**j**) mRNA expression in freshly isolated RPTs from WT control mice, *Hnrnpf*-Tg mice (F-Tg), Akita mice and Akita *Hnrnpf*-Tg mice (Akita F-Tg) at week 20. Values are mean + SEM corrected to β-actin, *n* = 6. *p < 0.05; **p < 0.01

Akita mice developed renal structural damage compared with WT and *Hnrnpf*-Tg mice (ESM Fig. 1a, PAS staining), including tubular luminal dilatation with accumulation of cell debris, increased extracellular matrix proteins in glomeruli and tubules, and proximal tubule cell atrophy. HnRNP F overexpression markedly reversed but never completely resolved these abnormalities in Akita mice. We detected significant increases in Masson’s trichrome staining (Fig. 3a) and immunostaining for collagen type IV (Fig. 3b), fibronectin 1 expression (Fig. 3c) and collagen type I (Fig. 3d) in glomerulotubular areas in Akita mice compared with WT controls and *Hnrnpf*-Tg mice. These changes were reduced in Akita *Hnrnpf*-Tg mice. Quantification of Masson’s trichrome-stained (ESM Fig. 1b), immunostaining of collagen IV (Fig. 3e), fibronectin 1 (Fig. 3f) and collagen I (Fig. 3g), and RT-qPCR quantification of mRNA levels (Fig. 3h–j) confirmed their expression.

### HnRNP F overexpression enhances *Ace-2* and suppresses *Agt*, *Tgf-β1* and *Tgf-βrII* gene expression and protein levels in rat RPTCs in vitro

**Fig. 4:**
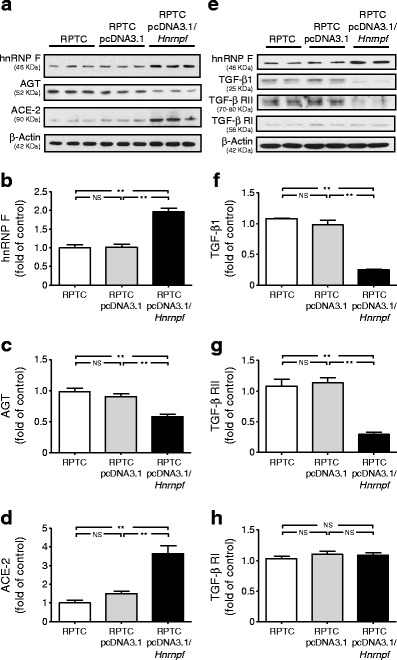
hnRNP F overexpression inhibits AGT, TGF-β1, TGF-β1 RII and enhances ACE-2 protein expression in RPTCs. Immunoblotting (**a**) and quantification of hnRNP F (**b**), AGT (**c**) and ACE-2 (**d**) protein levels by densitometry in naive RPTCs, RPTC-pcDNA 3.1 or RPTC-pcDNA 3.1/*Hnrnpf* after a 24 h culture. Immunoblotting (**e**) and quantification of TGF-β1 (**f**), TGF-β RII (**g**) and TGF-β RI (**h**) protein levels in rat RPTCs. Values, corrected to β-actin protein levels, are mean + SEM, *n* = 3. The experiments were repeated twice. **p < 0.01

RPTCs stably transfected with pcDNA 3.1/*Hnrnpf* (RPTC-pcDNA 3.1/*Hnrnpf*) exhibited considerably higher levels of hnRNP F (Fig. 4a,b), lower amounts of AGT (Fig. 4a,c) and a higher amount of ACE-2 (Fig. 4a,d) than non-transfected RPTCs or RPTCs stably transfected with pcDNA 3.1 (RPTC-pcDNA 3.1).

In contrast, TGF-β1 and TGF-β RII protein levels were significantly decreased in RPTC-pcDNA 3.1/*Hnrnpf* compared with non-transfected RPTCs or RPTC-pcDNA 3.1 (*p* < 0.01) (Fig. 4e,f,g, respectively). TGF-β RI protein level was similar in non-transfected RPTCs, RPTC-pcDNA 3.1 or RPTC-pcDNA 3.1/*Hnrnpf* (Fig. 4h).

RT-qPCR of *Hnrnpf*, *Agt*, *Ace-2*, *Tgf-β1*, *Tgf-βrII* and *Tgf-βrI* mRNA levels confirmed these findings (ESM Fig. 2a–f).

### TGF-β1 signalling and inhibition of *Ace-2* gene expression in rat RPTCs

**Fig. 5:**
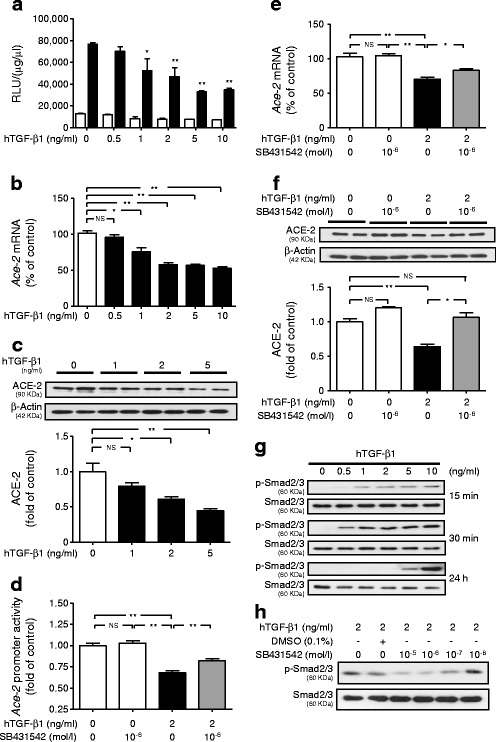
Human recombinant TGF-β1 inhibits *Ace-2* gene expression in rat RPTCs. TGF-β1 inhibits rat *Ace-2* gene promoter activity (**a**) (white bars, pGL4.20; black bars, pGL4.20-rat *Ace-2* promoter [N-1,091/+83]), *Ace-2* mRNA (**b**) and ACE-2 protein (**c**) expression in rat RPTCs in a dose-dependent manner. SB431542 (a specific TGF-β RI inhibitor) reversed the suppressive effect of TGF-β1 on *Ace-2* gene promoter activity (**d**), *Ace-2* mRNA (**e**) and ACE-2 protein (**f**) levels in rat RPTCs. TGF-β1 stimulated the phosphorylation of Smad2/3 in a dose- and time-dependent manner (**g**) and reversed it in the presence of SB431542 (**h**). Rat *Ace-2* gene promoter activity was measured by luciferase activity assay. Values are mean + SEM, *n* = 3. Similar results were obtained in three independent experiments. *p < 0.05; **p < 0.01, RLU, relative light units

TGF-β1 inhibited rat *Ace-2* gene promoter activity (Fig. 5a), rat *Ace-2* mRNA expression (Fig. 5b) and rat ACE-2 protein level (Fig. 5c) in a concentration-dependent manner, which was reversed by SB431542 (a TGF-β RI inhibitor) (Fig. 5d–f, respectively). Furthermore, TGF-β1 stimulated Smad 2/3 phosphorylation in a concentration- and time-dependent manner (Fig. 5g) and reversed by SB431542 (Fig. 5h). These data demonstrate that TGF-β1 inhibition of *Ace-2* gene transcription is mediated, at least in part, via Smad2/3 signalling.

### HnRNP F overexpression prevents TGF-β signalling, and TGF-β inhibition of *Ace-2* and induction of fibrotic gene expression in RPTCs

**Fig. 6:**
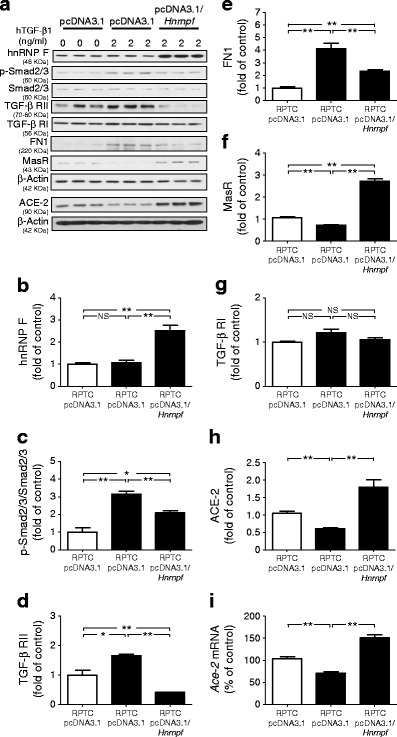
hnRNP F overexpression prevents TGF-β1 signalling, stimulation of profibrotic gene and inhibition of ACE-2 expression in rat RPTCs. (**a**) Immunoblotting of hnRNP F, Smad2/3 phosphorylation, TGF-β RII, TGF-β RI, fibronectin 1 (FN1), MasR and ACE2 levels in naive RPTCs, RPTC-pcDNA 3.1 or RPTC-pcDNA 3.1/*Hnrnpf* in the presence or absence of TGF-β1 (2 ng/ml) after 24 h culture. Quantification of the level of hnRNP F (**b**), Smad2/3 phosphorylation (**c**), TGF-β RII (**d**), fibronectin 1 (**e**), MasR (**f**), TGF-β RI (**g**), ACE-2 (**h**) and *Ace-2* mRNA (**i**). Values are mean + SEM, *n* = 3. Similar results were obtained in three independent experiments. *p < 0.05; **p < 0.01

TGF-β1 had no detectable effect on hnRNP F protein levels (Fig. 6a,b). Intriguingly, hnRNP F overexpression prevented TGF-β1 stimulation of Smad 2/3 phosphorylation (Fig. 6a,c), TGF-β RII expression (Fig. 6a,d) and fibronectin 1 expression (Fig. 6a,e). HnRNP F overexpression also prevented TGF-β1-induced downregulation of MasR (Fig. 6a,f) content in RPTCs. Addition of TGF-β1 did not affect TGF-β RI expression in RPTCs (Fig. 6a,g).

Furthermore, overexpression of hnRNP F prevented the inhibitory effect of TGF-β1 on ACE-2 protein (Fig. 6a,h) and *Ace-2* mRNA (Fig. 6i) expression in RPTC-pcDNA 3.1/*Hnrnpf*.

### Localisation of *Hnrnpf*- and TGF-β1 (or SMAD)-RE in rat *Ace-2* gene promoter

**Fig. 7:**
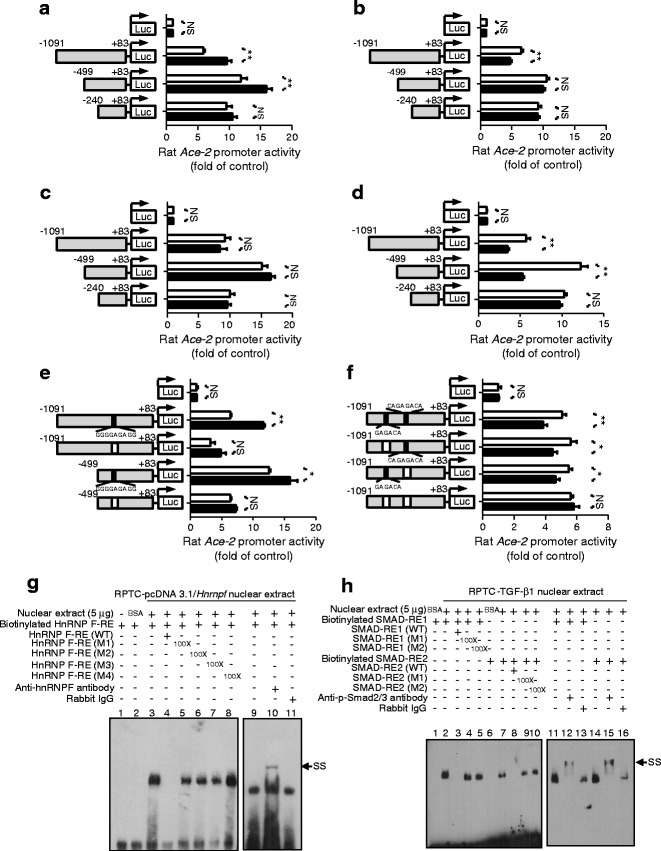
Identification of *Hnrnpf-RE* and *SMAD-RE* in the *Ace-2* gene promoter. (**a**) Luciferase activity of the plasmid containing various lengths of *Ace-2* gene promoter in RPTC-pcDNA 3.1 (white bars) and in RPTC-pcDNA 3.1/*Hnrnpf* (black bars); (**b**) in RPTC-pcDNA 3.1 ± TGF-β1 (white bars, without hTGF-β1; black bars, with 2 ng/ml hTGF-β1); and (**c**) in RPTC-pcDNA3.1/*Hnrnpf* ± TGF-β1 (white bars, without hTGF-β1; black bars, with 2 ng/ml hTGF-β1); (**d**) in RPTC-pcDNA 3.1 ± *Hnrnpf* siRNA (white bars, treated with 50 nmol/l scrambled siRNA; black bars, treated with 50 nmol/l *Hnrnpf* siRNA), cultured in normal glucose media for 24 h. (**e**) Promoter activity of the *Ace-2* gene ± *Hnrnpf-RE* in RPTC-pcDNA 3.1 (white bars) and in RPTC-pcDNA 3.1/*Hnrnpf* (black bars) or (**f**) ± *SMAD-RE*s in RPTC-pcDNA 3.1 in the absence or presence of TGF-β1 (white bars, without hTGF-β1; black bars, with 2 ng/ml hTGF-β1). Values are mean + SEM, *n* = 6. The experiments were repeated twice. *p < 0.05; **p < 0.01. EMSA and supershift EMSA of the putative biotinylated *Hnrnpf-RE* (**g**) and biotinylated *SMAD-REs* (**h**) with RPTC nuclear proteins ± excess unlabelled WT *Hnrnpf-RE* or mutated *Hnrnpf-REs* (M1 to M4 are mutants of *Hnrnpf-RE* with nucleotides mutated or deleted in the binding motif as shown in ESM Table 2) or WT *SMAD-RE* or mutant *SMAD-REs* (*SMAD-RE1* [M1 and M2] and *SMAD-RE2* [M1 and M2] are mutants of respective *SMAD-RE1* and *SMAD-RE2* with nucleotides mutated in the binding motif as shown in ESM Table 2). Rabbit IgG or rabbit anti-hnRNP F or anti-Smad2/3 antiserum was added to the reaction mixture and incubated for 30 min on ice before incubation with the biotinylated probe. Results are representative of three independent experiments. SS, supershift band

To localise the putative DNA-RE(s) that mediate(s) the action of hnRNP F or TGF-β1 on *Ace-2* gene promoter activity, plasmids containing various lengths of the rat *Ace-2* gene promoter were transiently transfected into RPTC-pcDNA 3.1 or RPTC-pcDNA 3.1/*Hnrnpf*. The activity of pGL4.20-*Ace-2* promoter (N-1,091/+83) and pGL 4.20-*Ace-2* promoter (N-499/+83) exhibited respective fivefold and 12-fold increase as compared with the control plasmid, pGL 4.20 in RPTC-pcDNA 3.1 (Fig. 7a). Further deletion of nucleotides N-499 to N-241 (pGL 4.20-*Ace-2* promoter [N-240/+83]) significantly reduced the rat *Ace-2* promoter activity. Moreover, the activity of pGL4.20-*Ace-2* promoter (N-1,091/+83) and pGL4.20-*Ace-2* promoter (N-499/+83) was further increased by 1.5–2.0-fold, whereas the activity of pGL4.20-*Ace-2* promoter (N-240/+83) did not increase in RPTC-pcDNA 3.1/*Hnrnpf* as compared with RPTC-pcDNA 3.1 (Fig. 7a). Interestingly, addition of TGF-β1 inhibited the promoter activity of pGL 4.20-*Ace-2* promoter (N-1,091/+83) and did not affect the activity of pGL 4.20-*Ace-2* promoter (N-499/+83) and pGL 4.20-*Ace-2* promoter (N-240/+83) in RPTC-pcDNA 3.1 (Fig. 7b). However, TGF-β1 had no inhibitory effect on the promoter activity of these constructs in RPTC-pcDNA 3.1/*Hnrnpf* (Fig. 7c).

In contrast, transfection of *Hnrnpf* siRNA significantly inhibited the promoter activity of pGL 4.20-*Ace-2* promoter (N-1,091/+83) and pGL 4.20-*Ace-2* promoter (N-499/+83) without affecting the activity of pGL 4.20-*Ace-2* promoter (N-240/+83) in RPTC-pcDNA 3.1 (Fig. 7d). Deletion of the nucleotides N-401 to N-393 (5′-ggggagagg-3′) in the *Ace-2* gene promoter markedly attenuated the promoter activity of pGL 4.20-*Ace-2* promoter (N-1,091/+83) and pGL 4.20-*Ace-2* promoter (N-499/+83) in RPTC-pcDNA 3.1/*Hnrnpf* (Fig. 7e). Interestingly, deletion of the putative proximal *SMAD-RE* (nucleotides N-511 to N-504 [5′-cagagaca-3′]) or distal putative *SMAD-RE2* (nucleotides N-789 to N-784 [5′-gagaca-3′]) in the *Ace-2* gene promoter partially attenuated whereas deletion of both *REs* (nucleotides N-511 to N-504 and nucleotides N-789 to N-784) completely abolished the inhibitory action of TGF-β1 on pGL 4.20-*Ace-2* promoter (N-1,091/+83) activity in RPTC-pcDNA 3.1 (Fig. 7f). Furthermore, EMSA showed that the double strand DNA fragments, nucleotides N-405 to N-387 (putative *Hnrnpf-RE*), nucleotides N-518 to N-497 (putative proximal *SMAD-RE1*) and nucleotides N-797 to N-776 (putative distal *SMAD-RE2*) bind to the nuclear proteins from RPTCs and they could be displaced by the respective WT DNA fragments, but not by mutated DNA fragments (Fig. 7g,h, respectively). Importantly, addition of anti-hnRNP F and anti-Smad 2/3 antibody induced a supershift of the respective *Hnrnpf-RE* and *SMAD-REs* with the nuclear proteins, respectively (Fig. 7g,h).

## Discussion

The present report identifies a novel mechanism by which hnRNP F prevents hypertension and kidney injury in diabetic Akita mice, i.e. hnRNP F stimulation of renal *Ace-2* gene transcription and mitigation of the inhibitory effect of TGF-β1 on *Ace-2* gene transcription.

We reported previously that overexpression of hnRNP F prevents systemic hypertension, and inhibits renal *Agt* gene expression and RPTC hypertrophy in diabetic Akita *Hnrnpf*-Tg mice [29]. The present paper provides new in vivo and in vitro evidence that hnRNP F stimulates *Ace-2* gene transcription via binding to the DNA-RE of the *Ace-2* gene promoter, which is critical for the formation of renal *Ang 1–7* and subsequent expression of its antihypertensive and renoprotective actions in Akita mice [37].

HnRNP F, a member of the pre-mRNA-binding protein family [38] regulates gene expression at both the transcriptional and post-transcriptional levels. Indeed, hnRNP F engages in alternative splicing of various genes [39–41] and associates with TATA-binding protein, RNA polymerase II, nuclear cap-binding protein complex and various transcriptional factors.[42, 43]

The Akita mouse is an autosomal-dominant model of spontaneous type 1 diabetes in which the *Ins2* gene is mutated. Akita mice develop hyperglycaemia and systemic hypertension, leading to cardiac hypertrophy, left ventricular diastolic dysfunction, glomerulosclerosis and enhanced oxidative stress in RPTs, closely resembling those observed in patients with type 1 diabetes [44, 45].

Our study provides evidence for a novel mechanism for hnRNP F lowering of SBP: inhibition of intrarenal *Agt* gene expression and RAS activation, concomitant with upregulation of the ACE-2/Ang 1–7/MasR axis. Indeed, our results show that hnRNP F overexpression inhibited renal AGT and *Agt* mRNA expression (ESM Fig. 1 c–e), lowered urinary AGT and Ang II levels and normalised urinary Ang 1–7 levels.

We consistently observed decreased renal ACE-2 expression in Akita mice as previously reported [23, 24]. Decreased ACE-2 expression also has been reported in male streptozotocin (STZ)-induced diabetic mice [46], STZ-induced diabetic rats [47, 48] and human type 2 diabetic kidneys [49, 50].

The precise mechanism by which hnRNP F overexpression leads to upregulation of renal *Ace-2* and *MasR* gene expression in diabetes remains unclear. One possibility is that hnRNP F binds to putative *Hnrnpf-RE(s)* in the *Ace-2* and *MasR* gene promoters, subsequently enhancing *Ace-2* and *MasR* gene transcription. This possibility is supported by our findings that hnRNP F considerably augments the activity of an *Ace-2* gene promoter and that the *Hnrnpf* siRNA and deletion of the putative *Hnrnpf-RE* markedly reduced the rat *Ace-2* gene promoter activity in RPTCs. Furthermore, the biotinylated-labelled *Hnrnpf-RE* specifically bound to RPTC nuclear proteins and the addition of anti-hnRNP F antibody yielded a supershift of biotinylated-labelled *Hnrnpf-RE* binding with nuclear proteins in EMSA. These data demonstrate that hnRNP F binds to the putative *Hnrnpf-RE* and stimulates *Ace-2* gene transcription. Of note, hnRNP F is not specific for *Ace-2* gene expression but also affects the expression of *Agt* [25] and other genes [51, 52].

Currently, little is known about the mechanisms by which TGF-β1 downregulates renal *Ace-2* gene expression in diabetes. Chou et al [53] reported that SB431542 inhibited high glucose and TGF-β1 inhibition of *Ace-2* mRNA expression in cultured NRK-52 cells. Our findings confirm these observations. Our present studies also demonstrate that TGF-β1 inhibits the activity of pGL 4.20-rat *Ace-2* promoter (N-1,091/+83) and that deletion of putative *SMAD-REs* in the *Ace-2* gene promoter mitigates the inhibitory effect of TGF-β1 on the *Ace-2* gene promoter activity. Furthermore, biotinylated-labelled *SMAD-REs* bound to RPTC nuclear proteins and the addition of anti-Smad2/3 antibody yielded a supershift of labelled DNA with nuclear proteins. These data demonstrate that the inhibitory effect of TGF-β1 on *Ace-2* gene transcription is mediated, at least in part, via the *SMAD-REs* in the *Ace-2* gene promoter.

Intriguingly, hnRNP F overexpression prevented TGF-β1 signalling on Smad2/3 phosphorylation and on TGF-β1 inhibition of *Ace-2* gene promoter activity in RPTCs. At present, the underlying molecular mechanism of how hnRNP F prevents TGF-β1 inhibition of *Ace-2* gene transcription is not yet defined. One possibility might be that hnRNP F directly inhibits *Tgf-β1rII* gene expression as shown in our studies. The second possibility is that hnRNP F might interfere or prevent the interaction of Smad2/3 with other transcriptional factor(s) to inhibit *Ace-2* gene transcription. Clearly, more studies are needed to define the molecular interaction of hnRNP F with Smad2/3 on *Ace-2* gene transcription.

In summary, the present study suggests a major role for hnRNP F in attenuating systemic hypertension and renal fibrosis in experimental diabetes and possibly in diabetic human kidneys. Our observations raise the possibility that selective targeting of this antihypertensive and anti-fibrotic protein may represent a novel approach for preventing or reversing the pathological manifestations of DN, particularly tubular fibrosis.

## Electronic supplementary material

Below is the link to the electronic supplementary material.

ESM Fig. 1 (PDF 307 kb)

ESM Fig. 2 (PDF 114 kb)

ESM Table 1 (PDF 45 kb)

ESM Table 2 (PDF 85 kb)

## Abbreviations

ACR: Albumin/creatinine ratio
AGT: Angiotensinogen
Ang: Angiotensin
BW: Body weight
DN: Diabetic nephropathy
EMSA: Electrophoretic mobility shift assay
ESRD: End-stage renal disease
hnRNP F: Heterogeneous nuclear ribonucleoprotein F
KAP: Kidney-specific androgen-regulated protein
KW: Kidney weight
MasR: Angiotensin 1–7 receptor
RAS: Renin–angiotensin system
RE: Response element
ROS: Reactive oxygen species
RPTs: Renal proximal tubules
RPTCs: Renal proximal tubular cells
RT-qPCR: Real-time-quantitative PCR
SBP: Systolic BP
siRNA: Small interfering RNA
STZ: Streptozotocin
Tg: Transgenic
TL: Tibial length
TGF-β RI(RII): TGF-β receptor I(II)
WB: Western blotting
WT: Wild-type
