# Supplementary material for: Overexpression of heterogeneous nuclear ribonucleoprotein F stimulates renal Ace-2 gene expression and prevents TGF-β1-induced kidney injury in a mouse model of diabetes
Source: Diabetologia. 2015 Aug 1;58(10):2443–54. doi: 10.1007/s00125-015-3700-y (PMC4572079; doi:10.1007/s00125-015-3700-y)
Supplement: Supplementary file 1 — (PDF 307 kb) [file 125_2015_3700_MOESM1_ESM.pdf]

ESM Fig. 1

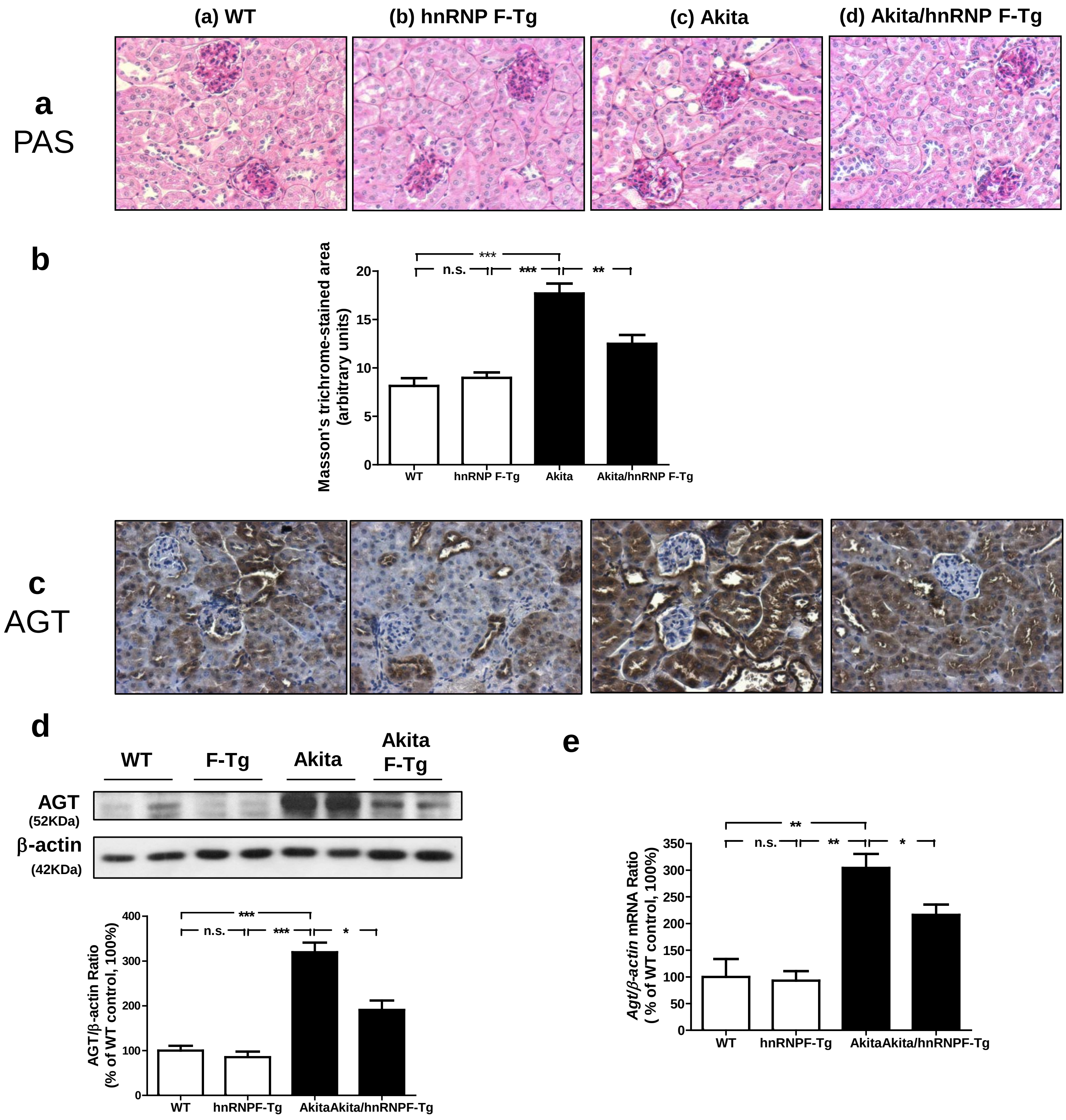

ESM Fig. 1. (a) Periodic acid Schiff (PAS) staining in kidneys of WT control, hnRNP-Tg, Akita and Akita hnRNP F-Tg mice. (b) Semi-quantitative analysis of Masson's trichrome staining in glomerulo-tubular areas of kidneys of WT control, hnRNP-Tg, Akita and Akita hnRNP F-Tg mice. Immuno-histochemical staining (c), Western blotting (d) and qPCR (e) of Agt expression in kidneys of WT control, hnRNP-Tg, Akita andAkita hnRNP F-Tg mice. Values are mean+SEM corrected to β-actin, n=6. (*$p<0.05$; **$p<0.01$; NS, not significant).
