# Supplementary material for: Overexpression of heterogeneous nuclear ribonucleoprotein F stimulates renal Ace-2 gene expression and prevents TGF-β1-induced kidney injury in a mouse model of diabetes
Source: Diabetologia. 2015 Aug 1;58(10):2443–54. doi: 10.1007/s00125-015-3700-y (PMC4572079; doi:10.1007/s00125-015-3700-y)
Supplement: Supplementary file 2 — (PDF 114 kb) [file 125_2015_3700_MOESM2_ESM.pdf]

ESM Fig. 2

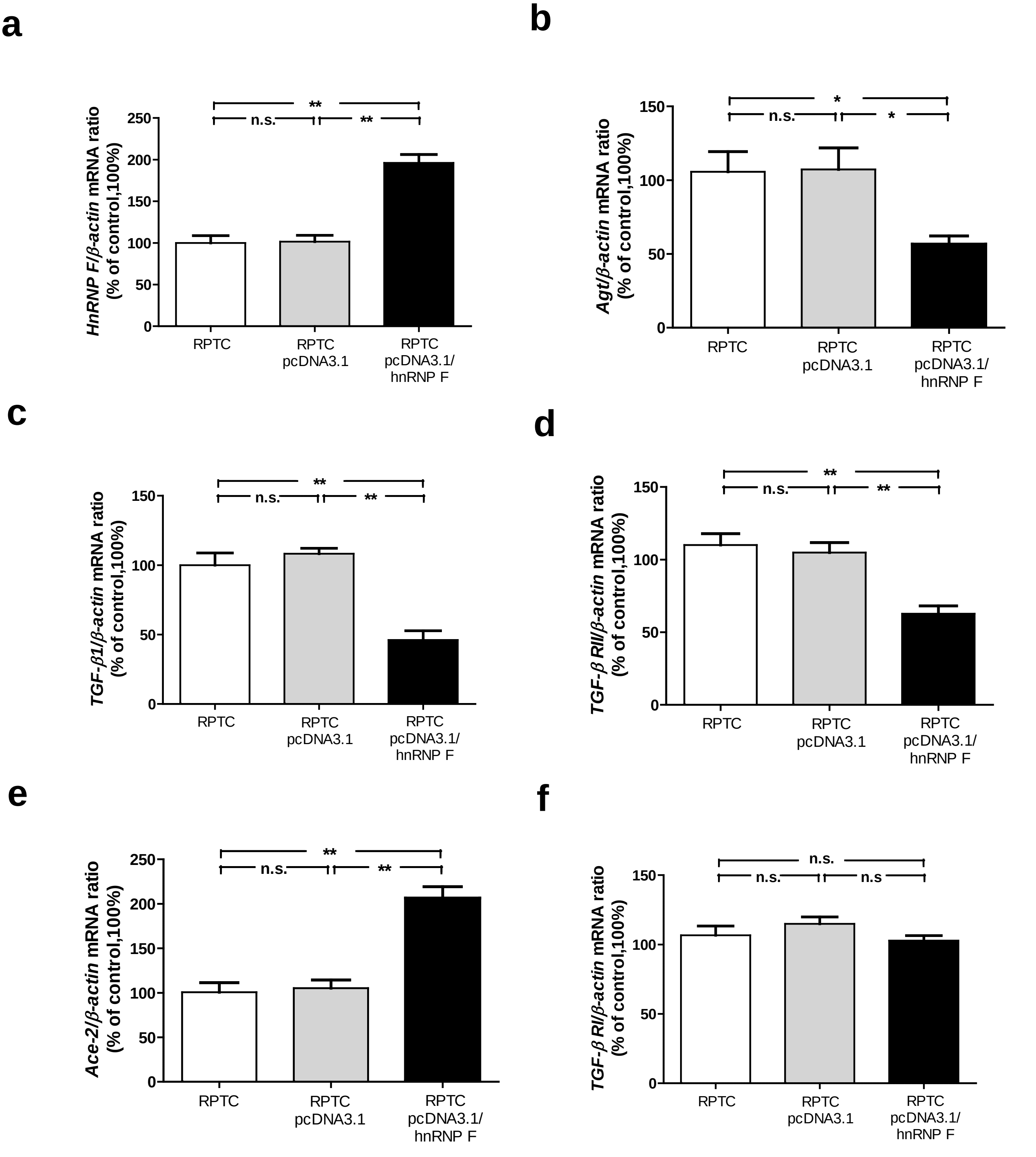

ESM Fig. 2. HnRNP F overexpression (a) inhibits AGT (b), TGF-β1 (c), TGF-β1 RII (d) and enhances ACE-2 (e) but has no effect on TGF-β1 RI (f) mRNA expression in RPTCs. Values, corrected to β-actin protein levels, are mean+SEM, n=3. The experiments were repeated twice. ((**p*<0.05; ***p*<0.01; NS, not significant).
