# Supplementary material for: Overexpression of heterogeneous nuclear ribonucleoprotein F stimulates renal Ace-2 gene expression and prevents TGF-β1-induced kidney injury in a mouse model of diabetes
Source: Diabetologia. 2015 Aug 1;58(10):2443–54. doi: 10.1007/s00125-015-3700-y (PMC4572079; doi:10.1007/s00125-015-3700-y)
Supplement: Supplementary file 3 — (PDF 45 kb) [file 125_2015_3700_MOESM3_ESM.pdf]

ESM Table 1 (Antibody)

Antibody	Host	Dilution for WB	Dilution for IHC	Supplier (Antibody validated by the supplier unless indicated)
hnRNP F	Rabbit polyclonal	1:10000	1:200	specifically recognizing hnRNP F (CTARRYIGIVKQAGLER) were generated and validated in our labo [25]
ACE-2	Goat polyclonal	1 :2000	1 :100	R&D Systems
MasR (MAS1)	Rabbit polyclonal	1 :2000	1 :100	Novus biologicals
ACE	Goat polyclonal	1:2000	1:100	Santa Cruz Biotechnology
TGFβ1	Rabbit polyclonal	1 :2000	1 :100	Santa Cruz Biotechnology
TGF beta Receptor II	Rabbit polyclonal	1:2000	1:100	Santa Cruz Biotechnology
TGF beta Receptor I	Rabbit polyclonal	1 :2000	1 :100	Novus biologicals
Collagen type IV	Mouse Monoclonal		1 :100	Millipore
Fibronectin I	Rabbit polyclonal	1 :2000	1 :100	Sigma-Aldrich
Collagen type I	Rabbit polyclonal		1 :100	Abcam
Agt	Rabbit polyclonal	1:2000	1:200	specifically recognizing Agt were generated and validated in our lab [1]
p-Smad 2/3	Rabbit polyclonal	1 :2000		Cell signaling
Smad 2/3	Rabbit polyclonal	1 :2000		Chemicon
β-Actin	Mouse Monoclonal	1 :20000		Sigma-Aldrich

1. Wang L, Lei C, Zhang SL et al. Synergistic effect of dexamethasone and isoproterenol on the expression of angiotensinogen in immortalized rat proximal tubular cells. Kidney international 1998; 53: 287-295
