# Supplementary material for: Overexpression of heterogeneous nuclear ribonucleoprotein F stimulates renal Ace-2 gene expression and prevents TGF-β1-induced kidney injury in a mouse model of diabetes
Source: Diabetologia. 2015 Aug 1;58(10):2443–54. doi: 10.1007/s00125-015-3700-y (PMC4572079; doi:10.1007/s00125-015-3700-y)
Supplement: Supplementary file 4 — (PDF 85 kb) [file 125_2015_3700_MOESM4_ESM.pdf]

ESM Table 2 (Primers)

Gene	Primer sequences	Species	Reference Sequence
Hnrnp f	S:AGAGTGACCGGAGAAGCTGA<br>AS:GCTCTCCAGGCCACTGTAAG	Mouse<br>Rat	NM_133834.2<br>NM_001037286.1
HA-tag	AS:GGCGTAGTCAGGCACGTCGT		
AKITA<br>(*Ins 2*)	S:TGCTGATGCCCTGGCCTGCT<br>AS:TGGTCCCACATATGCACATG	Mouse	NC_000073.6
Ace	S:GACCGGACAGCCCAAGTG<br>AS:AGCTTCTTTATGATCCGCTTGATG	Mouse	NM_207624.5
Ace	S:GAGCCATCCTTCCCTTTTTC<br>AS:GGCTGCAGCTCCTGGTATAG	Rat	NM_012544.1
Ace2	S:ATATGACTCAAGGATTCTGGG<br>AS:GCTGCAGAAAGTGACATGATT	Mouse	NM_001130513.1
Ace2	S:GCCCAAAAGATGAACGAGGC<br>AS:GACGCTTGATGGTCGCATTC	Rat	NM_001012006.1
MasR	S:GCATTCGTCTGTGCCCTTCT<br>AS:TTCCGTATCTTCACCACCAAGA	Mouse	NM_008552.4
MasR	S:TTGACAGCGGAGAAGAGAGTCA<br>AS:TCCGTATCTTCACCACCAAGATG	Rat	NM_012757.2
Tgf-β1	S:CCAAACTAAGGCTCGCCAGTC<br>AS:GGCACTGCTTCCCGAATGTC	Mouse	NM_011577.1
Tgf-β1	S:ATACGCCTGAGTGGCTGTCT<br>AS:TGGGACTGATCCCATTGATT	Rat	NM_021578.2
Tgf-β RI	S:ATTGCCAGGACCATTGTGTTAC<br>AS:TCTCTGCCTCTCGGAACCAT	Mouse	NM_009370.2
Tgf-β RI	S:ATTGCAAGGACCATTGTGCTAC<br>AS:TCTCTGCCTCTCGGAACCAT	Rat	NM_012775.2
Tgf-β RII	S:TCGTTCAAGCAGACGGATGT<br>AS:ATCTTCTCCTGGGAGCAGCT	Mouse	NM_009371.3
Tgf-β RII	S:AGTTTTGCGACGTGACACTG<br>AS:GGCATCTTCCAGAGTGAAGC	Rat	NM_031132.3
Col4α1	S:CATGTCCATGGCACCCATCT<br>AS:ATGGCCGGTGCTTCACAAAC	Mouse	NM_009931.2
Col4α1	S:CCATCTGTGGACCATGGCTT<br>AS:GCGAAGTTGCAGACGTTGTT	Rat	NM_001135009.1
FN1	S:GGCCTGAACCAGCCTACAG<br>AS:TGAGCTTAAAGCCAGCGTCA	Mouse	NM_010233.2
FN1	S:GGGAAGAAAAGGAGCCCAGG<br>AS:CCTCTTGCTCTTCCCGGTTT	Rat	NM_019143.2
Col1α1	S:ATCTCCTGGTGCTGATGGAC<br>AS:ACCTTGTTTGCCAGGTTCAC	Mouse	NM_007742.3
β-*actin*	S:ATGCCATCCTGCGTCTGGAC<br>AS:AGCATTTGCGGTGCACGATGG	Mouse<br>Rat	NM_007393.3<br>NM_031144.2
Rat *Ace2* promoter	S :CGAACGCGTCACGATCTCATGCCTATGG<br>S :AAGATCTCTGAGTTAGAGGCTAGCCTCAT<br>S :AAGGTACCTAACCAAATGTCCAAGTTTGA<br>AS : CTTTCCCCGTGCGCCAAGATCC	Rat	-1091<br>-499<br>-240<br>+83
Rat *Ace2* promoter Smad binding element 1 (GAGACA)	S:AATCAATTTTTTTTCGTTTTTAGGGTTTCTTT GTATAGCCCTAG<br>AS:CTAGGGCTATACAAAGAAACCCTAAAAACG AAAAAAAATTGATT		Site directed mutagenesis primers
Rat *Ace2* promoter Smad binding element 2	S:TGATCCCAGCACTCAGGAGGAGCAGATCTC TGAGTTAGAGGC		Site directed mutagenesis primers

ESM Table 2 (Primers)

(CAGAGACA)	AS:GCCTCTAACTCAGAGATCTGCTCCTCCTGA GTGCTGGGATCA		
Rat *Ace2* promoter hnRNPF binding element (GGGGAGAGG)	S: AAGGAAGAGAGAAAAAGAGGATGGATCGC<br>AS: GCGATCCATCCTCTTTTTCTCTCTTCCTT		Site directed mutagenesis primers
Hnrnpf-RE probe	S:AGAAAGGGGAGAGGAAGAGG<br>AS:CCTCTTCCTCTCCCCTTTCT		Biotinylated probe for EMSA
*SMAD-RE*1 probe	S:CGTTTTTAGAGACAGGGTTTCT<br>AS: AGAAACCCTGTCTCTAAAAACG		Biotinylated probe for EMSA
SMAD-RE2 probe	S:CAGGAGGCAGAGACAAGCAGAT<br>AS:ATCTGCTTGTCTCTGCCTCCTG		Biotinylated probe for EMSA
Hnrnpf-RE (WT)	S:AGAAAGGGGAGAGGAAGAGG<br>AS:CCTCTTCCTCTCCCCTTTCT		Competitor
Hnrnpf-RE (M1)	S:AGAAAGGaaAGAGGAAGAGG<br>AS:CCTCTTCCTCTttCCTTTCT		Competitor
Hnrnpf-RE (M2)	S:AGAAAGGGGAaAGGAAGAGG<br>AS:CCTCTTCCTtTCCCCTTTCT		Competitor
Hnrnpf-RE (M3)	S:AGAAAGGGGAGAaaAAGAGG<br>AS:CCTCTTttTCTCCCCTTTCT		Competitor
Hnrnpf-RE (M4)	S:AAGGAAGAGAGAAAAGAGGATGGATCGC<br>AS: GCGATCCATCCTCTTTTCTCTCTTCCTT		Competitor
SMAD-RE1 (WT)	S:CGTTTTTAGAGACAGGGTTTCT<br>AS:AGAAACCCTGTCTCTAAAAACG		Competitor
SMAD-RE1 (M1)	S : CGTTTTTAGActCAGGGTTTCT<br>AS : AGAAACCCTGagTCTAAAAACG		Competitor
SMAD-RE1 (M2)	S : CGTTTTTAGAGACtGGGTTTCT<br>AS : AGAAACCCaGTCTCTAAAAACG		Competitor
SMAD-RE2 (WT)	S:CAGGAGGCAGAGACAAGCAGAT<br>AS:ATCTGCTTGTCTCTGCCTCCTG		Competitor
SMAD-RE2 (M1)	S : CAGGAGGCActGACAAGCAGAT<br>AS : ATCTGCTTGTCagTGCCTCCTG		Competitor
SMAD-RE2 (M2)	S : CAGGAGGCAGActCAAGCAGAT<br>AS : ATCTGCTTGagTCTGCCTCCTG		Competitor
